# Trends in Compensation for Primary Care and Specialist Physicians After Implementation of the Affordable Care Act

**DOI:** 10.1001/jamanetworkopen.2020.11981

**Published:** 2020-07-28

**Authors:** Walter R. Hsiang, Cary P. Gross, Sean Maroongroge, Howard P. Forman

**Affiliations:** 1 Yale School of Medicine, Yale University, New Haven, Connecticut; 2 Yale School of Management, Yale University, New Haven, Connecticut; 3 Department of Internal Medicine, Yale School of Medicine, Yale University, New Haven, Connecticut; 4 Cancer Outcomes Public Policy and Effectiveness Research (COPPER) Center, Yale School of Medicine, Yale University, New Haven, Connecticut; 5 MD Anderson Cancer Center, Houston, Texas; 6 Department of Radiology, Yale School of Medicine, Yale University, New Haven, Connecticut; 7 Yale School of Public Health, Yale University, New Haven, Connecticut

## Abstract

This survey study uses data from the Medical Group Management Association’s voluntary physician compensation survey from 2008 to 2017 to examine trends in compensation for primary care and specialist physicians after passage of the Affordable Care Act.

## Introduction

When the Affordable Care Act (ACA) was passed, physicians were unsure how their salaries would be affected. Since the implementation of the ACA, numerous factors may have affected physician compensation, including increased emphasis on alternative payment models and discounted insurance payments from health exchanges. The ACA also included 2 temporary fee increases specifically for primary care physicians (PCPs): the 2013-2014 “Medicaid fee bump” and the 2011-2015 Primary Care Incentive Program.^1^ Given these factors and the 10th anniversary of the ACA, we sought to answer the following 2 concerns: (1) how overall physician compensation has changed and (2) how PCP compensation has changed relative to specialist compensation since the ACA was passed.

## Methods

To examine trends in physician compensation since the passage of the ACA, we calculated the inflation-adjusted change in physician compensation from 2008 to 2017 using the voluntary physician compensation survey conducted by the Medical Group Management Association (MGMA), which represents more than 20 000 physicians from private practices, hospitals, academic departments, and other organizations.^2^ To our knowledge, this survey is the largest of its kind in the United States. The MGMA sample is not a random sample of all physicians, as it tends to overrepresent physicians in larger medical groups, but it is the only nationally representative compensation survey with samples of all specialty types. We also calculated the change in the specialist premium, or the gap between compensation for primary and specialist care, during this period. This study received a non–human research exemption from the Yale School of Medicine Institutional Review Board and followed the American Association for Public Opinion Research (AAPOR) reporting guideline.

## Results

From 2008 to 2017, specialist compensation increased by a weighted mean (SD) of 0.6% (1.2%) per year, from $378 600 to $399 300, whereas primary care compensation increased by 1.6% (2.2%) per year, from $214 100 to $247 300 (Figure). The specialist premium declined during this period, from $164 500 in 2008 to $152 000 in 2017, or from 77% to 61%.

**Figure.**
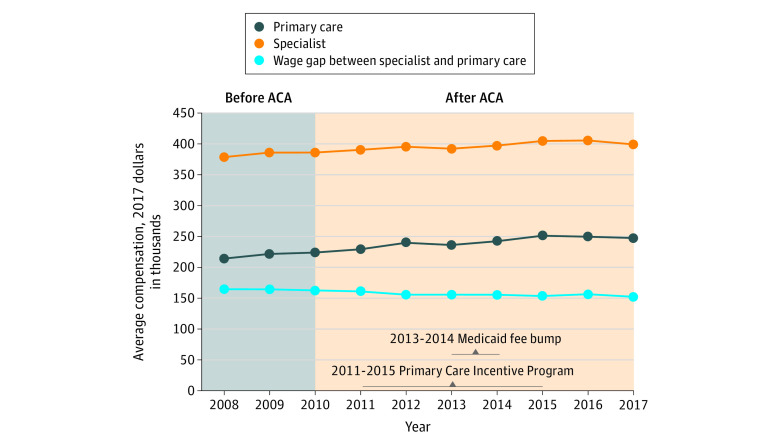
Weighted Mean Annual Compensation for Specialists and Primary Care Physicians From 2008 to 2017 ACA indicates Affordable Care Act.

## Discussion

Overall physician compensation has increased since the implementation of the ACA, with the growth in PCP compensation outpacing that of specialists. However, there continues to be a sizeable gap between compensation for primary and specialist care. More importantly, small changes to the specialty premium cannot necessarily be attributed to any specific policy or intervention.

Although the ACA has expanded insurance to millions of Americans, patients with Medicaid still face significant challenges accessing primary care appointments because of Medicaid’s low reimbursement levels.^3^ A straightforward financial adjustment to address this issue could entail a reinstatement of PCP fee increases. Private payers also could have a more active role in the future by either matching or administering Medicaid fee bumps in their own programs.

Because the opportunity cost of additional training for specialists is significant and the routine practice of specialist and primary care medicine is different, some specialist premium should be expected. In addition, while increasing PCP compensation might increase access to primary care, many patients with Medicaid will still face significant difficulty accessing specialty care. Regardless, primary care compensation in the era after passage of the ACA should consider the shifting role expected of PCPs, including increased management of midlevel health care professionals, such as physician assistants and nurse practitioners, and an increased patient caseload from expanded access to care.

A limitation of the MGMA sample was the overrepresentation of physicians from larger medical groups, which could overestimate the observed increases in compensation, because physicians from larger systems or hospitals tend to earn more money than those in private practice.^4^

In conclusion, this study found that compensation both for PCPs and for specialty physicians has increased since the ACA was implemented. Furthermore, the gap between specialty and primary care salaries remains sizeable. As we head into another cycle of potential major health care reform, policy makers should recognize that physician compensation will remain a significant concern and that differences in compensation between PCPs and specialists will matter.
